# Perceived social competencies as moderators: examining the relationship between psychological distress and aggression, hostility, and anger in Lebanese adults

**DOI:** 10.1186/s40359-024-01694-w

**Published:** 2024-04-17

**Authors:** Joya-Maria Karam, Zeinab Bitar, Diana Malaeb, Feten Fekih-Romdhane, Souheil Hallit, Sahar Obeid

**Affiliations:** 1 Faculty of Medicine, Lebanese University, Hadat, Lebanon; 2 Rennes University, Inserm, EHESP, Irset (Institut de recherche en santé, environnement et travail) - UMR_S 1085, F-35000 Rennes, France; 3 College of Pharmacy, Gulf Medical University, Ajman, United Arab Emirates; 4 The Tunisian Center of Early Intervention in Psychosis, Department of Psychiatry “Ibn Omrane”, Razi hospital, 2010 Manouba, Tunisia; 5 Faculty of Medicine of Tunis, Tunis El Manar University, Tunis, Tunisia; 6 School of Medicine and Medical Sciences, Holy Spirit University of Kaslik, Jounieh, P.O. Box 446, Lebanon; 7 Applied Science Research Center, Applied Science Private University, Amman, Jordan; 8 School of Arts and Sciences, Social and Education Sciences Department, Lebanese American University, Jbeil, Lebanon

**Keywords:** Perceived social competences, Psychological distress, Aggression, Hostility, Anger

## Abstract

**Introduction:**

The majority of people experience anger at some point in their lives when confronted with unpleasant situations. In social settings, anger can lead to aggressive and hostile in the absence of adequate social competences. Our study aims to examine the moderating role of perceived social competences in the association between psychological distress and anger expression (trait anger, hostility, physical aggression, and verbal aggression) among a sample of Lebanese adults.

**Methods:**

403 participants above 18 years (the mean age was 24.56 ± 8.46) were enrolled in the cross-sectional study with 73% female participants. The candidates were asked to complete a structured questionnaire including the following scales: (1) Buss–Perry Aggression Questionnaire-Short Form (BPAQ-SF), (2) the Depression Anxiety Stress Scale (DASS-8), (3) Perceived Social Competencies (PSC), and (4) The De Jong-Gierveld Loneliness Scale.

**Results:**

The interaction psychological distress by perceived social competence was not significantly associated with physical aggression, verbal aggression, or hostility but was significantly associated with anger. After adjusting the results over variables that showed a *p* <.25 in the bivariate analysis, this association was significant at low (Beta = 0.24; *p* <.001), moderate (Beta = 0.20; *p* <.001) and high (Beta = 0.16; *p* <.001) levels of perceived social competencies, where higher psychological distress was significantly associated with more anger. On another note, with higher perceived social competence, we find a decrease in levels of psychological distress in our sample.

**Conclusion:**

This study provided evidence that perceived social competencies such as communication skills, empathy and prosocial behaviors act as moderators in the association between psychological distress and anger. In future works, investigating and building advanced program in order to develop social competences of individuals might prove important. It is crucial to implement such strategies and projects in schools: this educational setting could be fruitful in a way that social skills could be instilled during childhood and anger-aggressive behaviors could be managed throughout adulthood.

## Introduction

Anger is a negatively toned emotion experienced by all individuals in the course of their lives [1]. In an evolutionary perspective, anger is a protective emotion that nurtures survival, which is an instinctual necessity for mankind. In this modern world, anger arousals are manifested by aggressive behaviors, particularly when its intensity overrides regulatory control mechanism [2]. Anger can then lead to a state of antagonism toward someone or something perceived as the source of aversive events [3]. In the past few decades, investigators have shown significant interest in exploring aggressive behaviors and anger especially in children and it has been acknowledged that anger expression comprises four key elements [4, 5]: the first components related to inflicting harm to others are physical aggression and verbal aggression [6]. The second component is hostility and lastly anger, which is also considered a trigger for aggression.

As for aggression, most of the theories are focused on explaining that aggressive behaviors are a “dynamic of communication”: it is a behavior aiming at inflicting physical damage to persons or properties (by hitting, punching, stabbing, shooting…) [5, 7]. These behaviors are manifested when the aggressor wants to cope with a situation perceived as stressful and threatening. In a recent statistical examination of murders in the USA [8], a number of 4,920 murderers was reported and aggressors were adults between the ages of 20 to 29 which was a significant count compared to other age groups. Moreover, a study conducted among 2,382 male respondents has concluded that untreated depression in men is associated with increased likelihood of developing negative male-typical behaviors such as “aggression” towards their spouse that can be physical or even verbal aggression and “substance abuse” [9]. Regarding hostility [10], it encompasses negative feelings and unfriendly attitude such as verbal or even non-verbal cues (body language, tone of voice, cold stares) towards peers that may fuel harmful and threatening actions. It is characterized [10] by feelings of resentment, cynicism and antagonism towards others. Interestingly, a recent study depicted an association between high personal distress scores and the use of hostile behavior. Scholars expanded the analysis upon explaining that hostility is often used as a “dysfunctional coping strategy” to avoid internalizing distress by projecting a negative attitude towards others [11]. The last component, which is anger, refers to the affective nature of aggression and it has been in-depth investigated by scholars. In 2015, the overall prevalence of “uncontrolled, intense and inappropriate” anger in USA has been reported in 7.8% of the population [12]. In a previous study designed to search for an association between psychological distress and anger, 422 participants of a Turkish university have been enrolled in this investigation [13]. Predictably, it suggests a strong and positive correlation between anger traits and psychological distress which is also a risk source. Another research [14] has studied anger traits among 45 myofascial pain patients: these patients have been assessed to have severe psychological distress symptoms such as depression, anxiety and emotional distress, the study then concluded that myofascial pain patients do experience significant levels of anger due to their constant distress and permanent pain; feelings of anger would be represented by gritting one’s teeth and contracting neck muscle. In summary, literature reviews have largely discussed the significant association between aggression, anger and hostility with psychological distress.

In order to regulate negative emotions and avoid intolerable behaviors due to distress, seminal contributions have been made by researchers that proposed a novel concept which is “social competences”. Perceived social competence or social intelligence [15, 16] has been defined as the ability of an individual to effectively engage in social interactions and communicate through many skills: (1) emotional regulation [17], which is the ability to acknowledge one’s emotions and to control them during stressful events; (2) self-awareness [18], which is defined by the capacity of someone to have reasonable judgment regarding a stressful situation; (3) self-esteem and self-confidence [19]. A meta-analysis [20] showed small to moderate decline in aggression with presence of social competences among school-aged children, and a similar study [21] concluded that aggression could be prevented by providing social-emotional competence in educational settings. A previous study involving 2265 Korean students [22] supported the moderating effect of social competency on aggression mediated by psychological distress. Investigators concluded that the higher the social competency level, the lower the level of aggressive behaviors through depression and emotional distress. It has been further explained [22] that improving emotional recognition in a social setting could moderate mental health and anger expression by avoiding stressful interpersonal situations and engaging in prosocial behaviors [11, 16] such as problem solving, listening, helping, and cooperating fruitfully. Moreover, a novel literature review [23] discussed the importance of using social skills (emotional regulation such as cognitive reappraisal) to have stronger engagement in society and accept social support in order to decrease psychological distress and attenuate adverse feelings (anger, hostility and aggression).

### Rationale of the study

The purpose of this paper is to contribute to the existing bank of findings regarding behavioral development of Lebanese adults. In fact, Lebanon is a lower-middle income Arab country of Middle East and North Africa (MENA) region that is subject to ongoing hassles and traumas affecting adults. In a world where distress has been proved to be prevalent in adults due to their exposure to extreme life stressors such as loss of job, financial obligations and chronic illnesses [24], Lebanon has been ranked as the second most stressed country and has been found to have the third-highest daily anger rate in the MENA region [25]. The nature of Lebanese lifestyle: stressful events and relationships’ problems designated Lebanon as an interesting target for the study of anger patterns in its population. Among Lebanese students, the prevalence of alexithymia [14], depression and aggression [26] has been found to be very high in comparison with students worldwide. On top of that, a recent study [27] reported that more than half of the Lebanese participants had depressive symptoms, anxiety and suicidal ideation associated with higher depression.

Mental health in Lebanon has been described as an “epidemic” [28]. Even before COVID-19, the economy dived into a financial crisis which disrupted the system of all sectors and until today, this situation has not ceased yet. In 2023, the World Bank [29] reported that the inflation in Lebanon has reached 171.2% which is one of the highest rates globally accompanied by the rise in prices of food and basic necessities for survival. On top of that, the Beirut port blast [30] was an unprecedented challenge at all levels; politics, social, economy, and most importantly inflicted traumas to many citizens. Due to these cumulative unfortunate events in Lebanon, a scarcity of studies examined aggressive behaviors among Lebanese adults and it has been strongly associated with psychological distress [28]. The unique and unprecedented stressors faced by population of this country may give rise to distinct patterns of anger and coping mechanisms. Understanding anger expression in our Lebanese’s sample is important to have an insight into the social-cultural aspect of anger in this country.

The present study was firstly motivated by the fact that no previous research focused on the moderating factors of personal factor in the association between psychological distress and anger expression in Lebanon. Secondly, there is no evidence-based reviews discussing perceived social competences as a moderating factor on aggressive behaviors in the MENA region although it has been largely elaborated in other countries [31, 32]. The uncompleted findings signal the need for additional studies to understand the adults’ perception of their social competences in order to regulate negative behaviors due to constant distress in Lebanon. The main objective of this study is to find a relationship between anger, aggression and hostility and psychological distress while identifying a moderating effect of perceived social competences. Given that loneliness has been considered a co-founding factor that push the individual to have negative perception of themselves, to have false convictions that they are not valued by their peers, and become subsequently aggressive and hostile towards others [7, 33], the present study proposes to adjust analyses over this variable. We hypothesized that aggression, hostility and anger will be positively correlated with psychological distress, and inversely correlated with perceived social competence.

## Methods

### Procedures

Utilizing Google Forms and an online consent process, a survey was formulated and distributed across various messaging platforms such as WhatsApp, Instagram, and Facebook Messenger. Employing a convenience sampling technique, 403 participants were enlisted between February and December 2022. To qualify for participation, individuals had to be Lebanese citizens residing in Lebanon and aged 18 or above. The project was promoted on social media platforms with a specified duration. Participation criteria encompassed being a resident and citizen of Lebanon of adult age. Upon giving digital consent, participants were instructed to respond to the aforementioned instruments, presented in a pre-randomized sequence to mitigate order effects, which took approximately 15 min. The survey ensured participant anonymity, and involvement was voluntary and unpaid. Participants were asked to fill a self-administered questionnaire created on Google form. Excluded were those who refused to fill out the questionnaire or were younger than 18 years old [26].

#### Minimal sample size

We used G*Power software v.3.0.10 to determine the sample size. The minimum required sample size was 318 participants, considering an alpha error of 5%, a power of 80%, a minimal model r-square of 5% and allowing 10 predictors to be included in the regression models.

#### Measures

*Buss–Perry Aggression Questionnaire-Short Form (BPAQ-SF)*. This questionnaire, validated in Lebanon [34], was used to assess the level of aggression of an individual [4, 26]. It is a self-report tool consisting of 29 items such as “If I have to resort to violence to protest my rights, I will”, “I have threatened people I know” and “When people annoy me, I may tell them what I think of them”. The BPAQ-SF yields 4 scales: (1) physical aggression (2), verbal aggression (3), anger and (4) hostility. The questionnaire should be answered on a 5-point Likert scale, ranging from 1 (extremely uncharacteristic of me) to 5 (extremely characteristic of me). Higher scores indicate higher aggressive behaviors [4]. Cronbach’s alpha values were as follows: physical aggression (0.73), verbal aggression (0.44), hostility (0.71) and anger (0.74).

*Depression Anxiety Stress Scale (DASS-8)*. The 8-items scale is used to screen psychological distress symptoms (depression, anxiety and stress). Each item is rated over a 4- point scale from 0 (did not apply to me at all) to 3 (applied to me very much) [35]. The total score of DASS-8 ranges from 0 to 24; the higher the score the higher the presence of psychological distress. It has been previously validated by an Arab example in the Middle Eastern region [36] and used in previous studies [37] (Cronbach’s alpha = 0.91).

*Perceived Social Competencies (PSC)*. PSC is a brief 5-item measure of social competence skills and prosocial behavior in children and adults [38]. The PSC measures the degree to which the individual is able to engage and maintain positive social interactions with others. Items include “I help other people” and “I ask others if I can be of help”. The response is made with a Likert-type scale of 1–5 (1 = Not true at all, 5 = Really true). Higher scores indicate higher social competence (Cronbach’s alpha = 0.87).

*The De Jong-Gierveld Loneliness Scale*. The 6-item De Jong Gierveld Loneliness Scale, validated in Arabic [39, 40], is a reliable and valid instrument designed to gauge an individual’s subjective experience of loneliness and social isolation. Participants rate these items using responses like “no,” “more or less,” and “yes,” with “more or less” consistently indicating a higher level of loneliness. To calculate the total score, using reverse coding we consider that 0,1 and 2 would respectively correspond to “no”, “more or less” and “yes”. The questionnaire comprises two sets of statements: three negatively phrased items and three positively framed items. To maintain consistency, this statement is reversely coded so that higher scores reflects greater loneliness. For example, if a statement was, " There are plenty of people that I can lean on in case of trouble”, individuals who strongly agree with this statement would likely score low on the loneliness scale, indicating lower levels of loneliness. This scale is highly reliable with a significant internal consistency (Cronbach’s coefficient = 0.81) [41].

### Demographics

Participants were asked to provide their demographic details consisting of age, sex, marital status, highest educational attainment, self-reported height and weight. Height and weight data were used to compute self-reported BMI as kg/m^2^ [42]. The physical activity index was calculated by multiplying its frequency (from 1 = *less than once a month* to 5 = *daily or almost daily*) by duration (from 1 = *under 10 min* to 4 = *over 30 min*) by strength (from 1 = *light* to 5 = *sustained heavy breathing and perspiration*) [43, 44]. Household Crowding Index (HCI), which is calculated by dividing the total count of people living in a household, excluding a new-born child, by the total number of rooms in that household, excluding the kitchen [45].

### Analytic strategy

No missing data was found in our database. The SPSS software v.26 was used to analyze the data. Composite reliability was assessed using Cronbach’s alpha, with values ≥ 0.70 considered adequate. All aggression variables showed a normal distribution (skewness and kurtosis between − 1 and + 1) [46] except for the physical aggression score. The log transformation was applied, which later showed to be normally distributed. The student t test was used to compare two means. The Pearson test was used to correlate two continuous variables. Four linear regressions were then conducted, taking each aggression variable as a dependent one. The moderation analysis was done using the PROCESS MACRO v.3.4, Model 1 (an SPSS add-on) [47]. Interaction terms were probed by examining the association of psychological distress with anger at the mean, 1 SD below the mean and 1 SD above the mean of the moderator (perceived social competence). Regression models were adjusted over variables that showed a *p* <.25 in the bivariate analysis. *P* <.05 was deemed statistically significant.

## Results

In the current cross-sectional study, the participants were 403 young adults among which we find more females (*n* = 294) than males (*n* = 109) with a female percentage of 73%. Our sample gathered data among a population with a mean age of 24.56 ± 8.46. Other characteristics of the sample can be found in Table 1.

**Table 1:** Sociodemographic and other characteristics of the participants (*n* = 403)

Variable	*n* (%)
Sex	
Male	109 (27.0%)
Female	294 (73.0%)
Marital status	
Single	339 (84.1%)
Married	64 (15.9%)
Education	
Secondary or less	25 (6.2%)
University	378 (93.8%)
	**Mean ± SD**
Age (in years)	24.56 ± 8.46
Physical activity index	26.81 ± 19.34
Household crowding index (persons/room)	1.09 ± 0.54
Body Mass Index (kg/m^2^)	25.04 ± 14.87
Physical aggression	4.97 ± 2.41
Verbal aggression	6.24 ± 2.44
Anger	6.73 ± 3.09
Hostility	6.75 ± 3.07
Loneliness	12.29 ± 5.07
Perceived social competence	21.90 ± 4.47
Psychological distress	9.21 6.35

### Association of factors with aggression variables

The bivariate analysis results are shown in Tables 2 and 3. A higher mean physical aggression score was significantly found in males compared to females (5.99 vs. 4.59; *p* <.001). More loneliness and psychological distress were significantly associated with physical (*r* =.18; *p* <.001 and *r* =.20; *p* <.001 respectively) and verbal aggression (*r* =.29; *p* <.001 and *r* =.29; *p* <.001 respectively), hostility (*r* =.34; *p* <.001 and *r* =.50; *p* <.001 respectively) and anger (*r* =.51; *p* <.001 and *r* =.57; *p* <.001 respectively). Older age was significantly associated with less hostility (*r* = −.11; *p* =.035). Finally, more perceived social competence was significantly associated with less verbal aggression (*r* = −.13; *p* =.008) and anger (*r* = −.16; *p* =.002).

**Table 2:** Bivariate analysis of categorical variables associated with the aggression scores

Variable	Physical aggression	Verbal aggression	Hostility	Anger
Sex				
Male	5.99 ± 3.11	5.95 ± 2.28	6.45 ± 3.09	6.34 ± 2.82
Female	4.59 ± 1.96	6.34 ± 2.49	6.86 ± 3.06	6.87 ± 3.18
*p*	**< 0.001**	0.145	0.237	0.123
Effect size	0.538	0.163	0.133	0.176
Marital status				
Single	4.93 ± 2.40	6.15 ± 2.36	6.82 ± 3.08	6.77 ± 3.13
Married	5.17 ± 2.46	6.69 ± 2.80	6.38 ± 3.01	6.50 ± 2.91
*p*	0.455	0.153	0.291	0.518
Effect size	0.093	0.208	0.144	0.089
Education				
Secondary or less	5.56 ± 3.27	5.84 ± 2.51	5.96 ± 2.44	5.56 ± 2.40
University	4.93 ± 2.34	6.26 ± 2.44	6.80 ± 3.10	6.81 ± 3.12
*p*	0.349	0.403	0.186	0.051
Effect size	0.221	0.169	0.301	0.449

Numbers in bold indicate significant *p* values

**Table 3:** Bivariate analysis of the continuous variables associated with the aggression scores

Variable	1	2	3	4	5	6	7	8	9	10	11
1. Physical aggression	1										
2. Verbal aggression	0.34***	1									
3. Hostility	0.41***	0.44***	1								
4. Anger	0.27***	0.44***	0.57***	1							
5. Loneliness	0.18***	0.29***	0.34***	0.51***	1						
6. Perceived social competence	− 0.06	− 0.13**	− 0.09	− 0.16**	− 0.11*	1					
7. Psychological distress	0.20***	0.29***	0.50***	0.57***	0.60***	− 0.09	1				
8. Age	0.05	0.04	− 0.11*	− 0.07	− 0.07	0.12*	− 0.07	1			
9. Body Mass Index	0.09	0.07	0.09	0.05	− 0.04	0.07	0.01	0.11*	1		
10. Household crowding index	− 0.02	− 0.04	0.02	0.05	0.06	− 0.04	0.14**	− 0.11*	− 0.07	1	
11. Physical activity index	0.09	− 0.04	0.02	− 0.002	− 0.14**	0.10*	− 0.03	0.14**	0.05	− 0.09	1

Numbers in the table reflect Pearson correlation coefficients; *p <.05; **p <.01; ***p <.001

### Multivariable analysis

Females had significantly less physical aggression than males (Beta = -1.50), whereas higher psychological distress (Beta = 0.07) was significantly associated with more physical aggression (Table 4, Model 1).

**Table 4:** Multivariable analyses

	Unstandardized Beta	Standardized Beta	*p*	95% CI
Model 1: Physical aggression as the dependent variable (Nagelkerke R^2^ = 0.139)				
Sex (females vs. males*)	-1.50	− 0.28	**< 0.001**	-2.02; − 0.99
Body Mass Index	0.01	0.05	0.285	− 0.01; 0.02
Physical activity	01	0.06	0.221	− 0.004; 0.02
Loneliness	0.05	0.10	0.080	− 0.01; 0.10
Perceived social competence	− 0.03	− 0.06	0.242	− 0.08; 0.02
Psychological distress	0.07	0.18	**0.003**	0.02; 0.11
Model 2: Verbal aggression as the dependent variable (Nagelkerke R^2^ = 0.134)				
Sex (females vs. males*)	0.16	0.03	0.552	− 0.36; 0.67
Marital status (married vs. single*)	0.69	0.10	**0.030**	0.07; 1.30
Body Mass Index	0.01	0.08	0.106	− 0.003; 0.03
Loneliness	0.09	0.18	**0.002**	0.03; 0.14
Perceived social competence	− 0.06	− 0.11	**0.022**	− 0.11; − 0.01
Psychological distress	0.07	0.17	**0.004**	0.02; 0.11
Model 3: Hostility as the dependent variable (Nagelkerke R^2^ = 0.270)				
Sex (females vs. males*)	− 0.04	− 0.01	0.900	− 0.64; 0.56
Education level (university vs. secondary or less*)	0.68	0.05	0.234	− 0.44; 1.81
Age	− 0.02	− 0.06	0.215	− 0.05; 0.01
Body Mass Index	0.02	0.11	**0.018**	0.004; 0.04
Loneliness	0.04	0.07	0.187	− 0.02; 0.11
Perceived social competence	− 0.04	− 0.05	0.236	− 0.09; 0.02
Psychological distress	0.22	0.45	**< 0.001**	0.16; 0.27
Model 4: Anger as the dependent variable (Nagelkerke R^2^ = 0.380)				
Sex (females vs. males*)	− 0.09	− 0.01	0.738	− 0.64; 0.45
Education level (university vs. secondary or less*)	1.13	0.09	**0.033**	0.09; 2.17
Age	0.01	0.02	0.682	− 0.02; 0.04
Loneliness	0.15	0.25	**< 0.001**	0.09; 0.21
Perceived social competence	− 0.07	− 0.10	**0.016**	− 0.12; − 0.01
Psychological distress	0.20	0.41	**< 0.001**	0.15; 0.25

Numbers in bold indicate significant *p* values

Married participants vs. single (Beta = 0.69), higher loneliness (Beta = 0.09) and psychological distress (Beta = 0.07) were significantly associated with more verbal aggression, whereas higher perceived social competence (Beta = − 0.06) was significantly associated with less verbal aggression (Table 4, Model 2).

Higher BMI (Beta = 0.02) and psychological distress (Beta = 0.22) were significantly associated with more hostility (Table 4, Model 3).

Having a university level of education compared to secondary or less (Beta = 1.13), higher loneliness (Beta = 0.15) and psychological distress (Beta = 0.20) were significantly associated with more anger, whereas higher perceived social competence (Beta = − 0.07) was significantly associated with less anger (Table 4, Model 4).

### Moderation analysis

**Fig. 1:**
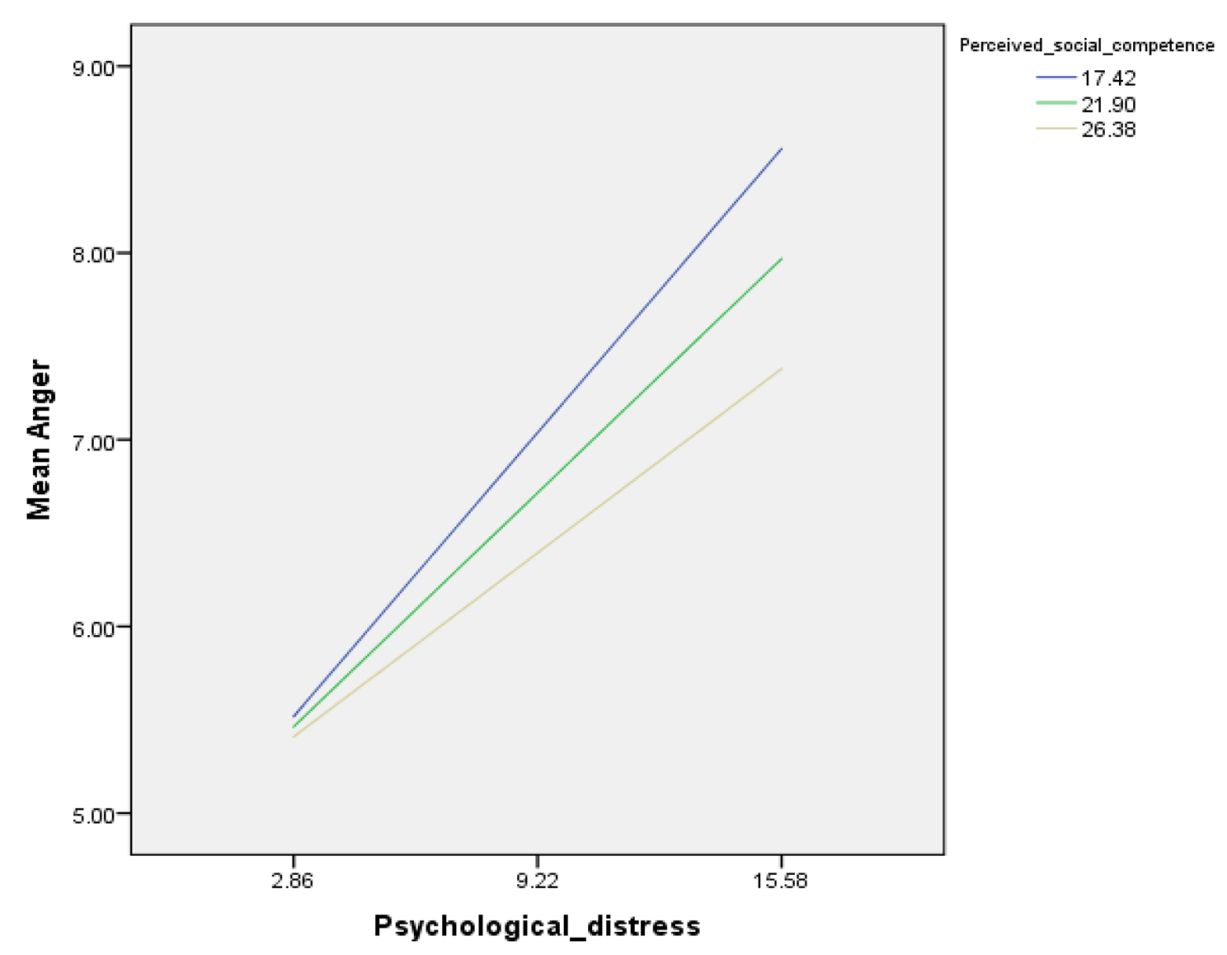
Association of the interaction psychological distress by perceived social competencies with anger

The interaction psychological distress by perceived social competence was not significantly associated with physical aggression, verbal aggression, or hostility but was significantly associated with anger (Table 5). After adjusting the results over variables that showed a *p* <.25 in the bivariate analysis, this association was significant at low (Beta = 0.24; *p* <.001), moderate (Beta = 0.20; *p* <.001) and high (Beta = 0.16; *p* <.001) levels of perceived social competencies, where higher psychological distress was significantly associated with more anger. On another note, with higher perceived social competence, we find a decrease in levels of psychological distress in our sample (Fig. 1; Table 6).

**Table 5:** Moderation analyses

	Beta	*t*	*p*	95% CI
Model 1: Physical aggression as the dependent variable (Nagelkerke R^2^ = 0.139)				
Psychological distress	0.08	0.91	0.361	− 0.09; 0.26
Perceived social competence	− 0.02	− 0.58	0.564	− 0.11; 0.06
Interaction content media exposure by perceived social competence	− 0.001	− 0.17	0.867	− 0.01; 0.01
Model 2: Verbal aggression as the dependent variable (Nagelkerke R^2^ = 0.134)				
Psychological distress	0.09	1.02	0.310	− 0.09; 0.27
Perceived social competence	− 0.05	-1.17	0.244	− 0.13; 0.03
Interaction content media exposure by perceived social competence	− 0.001	− 0.29	0.770	− 0.01; 0.01
Model 3: Hostility as the dependent variable (Nagelkerke R^2^ = 0.272)				
Psychological distress	0.31	2.97	**0.003**	0.11; 0.52
Perceived social competence	0.001	0.03	0.979	− 0.10; 0.10
Interaction content media exposure by perceived social competence	− 0.004	− 0.94	0.349	− 0.01; 0.01
Model 4: Anger as the dependent variable (Nagelkerke R^2^ = 0.386)				
Psychological distress	0.39	4.07	**< 0.001**	0.20; 0.58
Perceived social competence	0.01	0.17	0.865	− 0.08; 0.10
Interaction content media exposure by perceived social competence	− 0.10	-2.06	**0.040**	− 0.02; − 0.001

Numbers in bold indicate significant *p* values. Model 1 was adjusted over sex, body mass index, physical activity and loneliness. Model 2 was adjusted over sex, marital status, body mass index and loneliness. Model 3 was adjusted over sex, education level, age, body mass index and loneliness. Model 4 was adjusted over sex, education, age and loneliness

**Table 6:** Conditional effects of the focal predictor (psychological distress) on anger at values of the moderator (perceived social competence)

	Beta	*t*	*p*	95% CI
Low (= 17.43)	0.24	7.80	**< 0.001**	0.18; 0.30
Moderate (= 21.90)	0.20	8.31	**< 0.001**	0.15; 0.25
High (= 26.37)	0.16	5.22	**< 0.001**	0.10; 0.22

Numbers in bold indicate significant *p* values

## Discussion

The main aim of our study was to assess the association between psychological distress and anger expression while exploring the moderating role of perceived social competences. Our results showed that, after controlling for loneliness, the interaction psychological distress by perceived social competence was significantly associated with anger.

In presence of social competence, psychological distress was seen positively correlated to less anger traits in previous studies. Our study suggests that being equipped with robust social skills, self- awareness and problem-solving skills are contributing factors to decrease negative emotions induced by distress. Same results have been found by a previous experiment that [48] enrolled 128 students with moderate levels of anger and they were divided by a multi stage sampling process into an [1] experimental group and a [2] control group. Researchers found that scholars who developed problem solving skills and communication skills reported less anger levels compared to the control group. In line with our findings, another study [49] found that lower social competence scores were associated to higher levels of anger traits. It has been further explained that individuals with strong social competencies were less prone to provocation related to anger and subsequently to aggression in conflict settings. To prove the importance of developing social competences in order to manage anger attitudes related to psychological distress, literature reviews [50, 51] successfully implemented Social Skills Training (SST) as a treatment option for reinforcement of prosocial behavior to decrease risks of anger responses in social settings.

Extensive analysis carried out in our study showed that the interaction of psychological distress by perceived social competence was significantly associated with anger. This association was significant at low, moderate and high levels of perceived social competencies, where higher psychological distress was significantly associated with more anger. High level of psychological distress englobes many conditions such as depression and post-traumatic stress disorders (PTSD) that are hard to manage by personal self-assessment and intra-personal strategies [52] because exaggerated responses have been anchored in those who experience severe distress [53]. In fact, major traumas have been described to inflict greater long-term sequelae on the patient than a physical mechanical loss [54]. Therefore, people experiencing high levels of distress need to be closely followed up by healthcare professionals (psychiatrists, social workers…), social support [23] and sometimes they must be prescribed medications to regulate their mood and aggressive behaviors. Over time, extensive literature has established evidence-based treatment approaches [55] for high and complex emotional distress; a first line treatment would be cognitive behavioral therapy (CBT) that was proved to reduce the symptoms of PTSD more effectively than any other non-pharmaceutical treatment. In fact, high levels of distress may present with co-occurring disorders such as depression and substance abuse, which are anchored and grounded in patients, and perceived social skills may not provide individuals with adequate tools to cope with severe emotional distress. We interpret our finding by explaining that social competency is not an optimal protective factor able to manage aggressive behaviors during high distress situations. So, this makes it possible to think of further moderating factors that can better contribute to anger/ aggression management during high levels of distress.

Given the results of our study, we have to note that we did not reach the conclusion of a significant moderating effect of perceived social competences on the association between psychological distress and aggression (neither physical *p* =.972 or verbal aggression *p* =.630) and hostility (*p* =.325). These results could be interpreted by the fact that the Lebanese population is exposed to daily stressful events which makes it more difficult for them to constantly exert self-control and problem-solving skills to manage their anger. We speculate that this might be due to the dominance of female gender (73% of our sample size) among participants that are less likely to use aggression as a tool to externalize their anger and negative emotions [56]. We must acknowledge that there is a gender imbalance in the sample that could have led the study into a selection bias that interferes with our results. In fact, studies show [50] that men engage in more direct and physical aggression, and women tend to express aggression in more indirect and relational aggression. Therefore, future studies should aim to replicate results by carefully addressing the need for gender balance in the sample taken.

Alternatively, it might be related to uncovered confounding factors that decreased the likelihood of Lebanese adults resorting to aggression in stressful situation. A previous study [57] conducted among 252 Lebanese university found that aggressive behavior is negatively correlated to Emotional intelligence: Sociability, Self-control, Emotionality and Well-being. Under certain assumptions, we can consider that among our sample we might find other personal factors that led the participants to avoid using aggression during stressful events. In contrary, another study [1] found a positive correlation between association of perceived social competences and psychological distress with aggressive behaviors in a sample of substance abuse patients. In their longitudinal study, the researchers incited the intervention group to undergo 12 educational sessions for development of social competences (anger management, recognizing anger, communication skills, cognitive behavioral strategies…). This intervention resulted in decreasing the mean level of aggression from 54.11 to 47.72 after completion [1]. In line with this study, a similar approach [58] conducted among nineteen public Child care Centers recruited a sample of 361 children and demonstrated that social skills training was effective in decreasing aggressive behaviors in the treated group compared to the control group with a benefit rate higher in girls than in boys.

### Clinical implications

Nowadays, adult patients are suffering from severe anxiety, anger and aggression due to exposure to psychological distress and at the same time, healthcare providers are always in a continuous quest for knowledge to find new ways to help distressed patients. They would rather encourage the patient to pursue an aggression-anger management program [2, 48] in order to improve problem solving skills and communication skills than prescribing medications. These anger management therapies have been shown to decrease aggression [59, 60]and increase self-esteem and self-awareness of the individual [60]. This type of program should be constructively provided by a psychologist or a social worker: it consists of teaching [1] communication skills by encouraging patients to express their feelings, needs and boundaries in a constructive way [2], anger awareness and monitoring by inciting patients to identify their anger triggers, physical changes and behavioral responses and also [3] developing their problem-solving skills. The healthcare will have the objective to give the patient adequate tools in order to cope with aggression mediated by emotional distress. More importantly, in cases of underlying psychiatric disorders contribute to anger and aggression, healthcare may prescribe medications such as antidepressants, mood stabilizers or antipsychotics [61, 62] to reduce anger-state as a coadjutant treatment to psychotherapy in order to insure lasting changes in violent people. Nonetheless, the healthcare provider must be aware that development of social competences is not enough to reduce anger among patients with high distress levels (e.g., severe depression and PTSD), therefore other management strategies must be employed to assist the distressed patients.

### Limitations

This study holds several limitations. The data analyzed was collected via a self-reported questionnaire, which can affect the accuracy of the results because answers about perceived social competences and psychological distress may be underestimated or overestimated depending on the individuals’ mood while completing the questionnaire. For example, a previous study explained that people who have high levels of distress would exert more biases in their responses then people who report low levels of distress [31]. In addition, the convenience sampling technique used in this study and the unknown refusal rate can lead to sampling bias because the participants are being recruited based on referrals that may belong to a homogenous network and the researcher has limited control over the sampling process. Therefore, the recruited may not be representative of the entire population of interest. Moreover, some information like the place of residency (rural/urban) was not collected. Finally, it is crucial to recognize the plausible implication of other confounding factors, such as religiosity, social support and emotional intelligence that could potentially be “moderators” of anger. Looking forward, further attempts could provide better understanding whether there are other factors that could “moderate” anger.

In addition, we can mention that the verbal aggression subscale showed low reliability which can indicate low internal consistency for this scale and may be less precise in representing participants’ actual levels of verbal aggression. Moreover, the cross-sectional study of our study makes it difficult to rely on the obtained results because moods and emotions can change throughout life events and different periods of someone’s life. Therefore, a longitudinal study may accurately define the moderating effect of social competences on psychological distress and anger expression. It can be argued that a high rate of university students (93.8%) enrolled can lead to a selection bias that could unable the generalizability of our sample and restrict our findings to individuals with similar educational background. Moreover, the dominance of female gender in this study (73% of the sample) made it challenging to assess the association between psychological distress and aggression. This is why a more equally distributed sample would guarantee more accurate results.

## Conclusion

In conclusion, perceived social competences has been found to moderate the association between psychological distress and anger among Lebanese adults. Conversely, perceived social competences has not been found to have a moderating effect in the association of psychological distress and hostility and aggression. Moreover, this paper concluded by arguing that high perceived social competency was not a significant protective factor in order to control anger levels with presence of high distress level. Therefore, it would be highly interesting to assess the role of other moderators that could decrease the anger expression among the Lebanese population, such as religiosity, social support, and emotional intelligence. In future work, investigating and building advanced program in order to develop social competences of individuals might prove important. It is crucial to implement such strategies and project in schools: this educational setting could be fruitful in a way that social skills could be instilled during childhood and anger-aggressive behaviors could be managed throughout adulthood.

## Data Availability

All data generated or analyzed during this study are not publicly available due the restrictions from the ethics committee, but are available upon a reasonable request from the corresponding author.
